# SCANet: Implementation of Selective Context Adaptation Network in Smart Farming Applications

**DOI:** 10.3390/s23031358

**Published:** 2023-01-25

**Authors:** Xanno Sigalingging, Setya Widyawan Prakosa, Jenq-Shiou Leu, He-Yen Hsieh, Cries Avian, Muhamad Faisal

**Affiliations:** Department of Electronic and Computer Engineering, National Taiwan University of Science and Technology, Taipei City 10607, Taiwan

**Keywords:** deep learning, Selective Context Adaptation, smart farming, precision agriculture, level-wise information

## Abstract

In the last decade, deep learning has enjoyed its spotlight as the game-changing addition to smart farming and precision agriculture. Such development has been predominantly observed in developed countries, while on the other hand, in developing countries most farmers especially ones with smallholder farms have not enjoyed such wide and deep adoption of this new technologies. In this paper we attempt to improve the image classification part of smart farming and precision agriculture. Agricultural commodities tend to possess certain textural details on their surfaces which we attempt to exploit. In this work, we propose a deep learning based approach called Selective Context Adaptation Network (SCANet). SCANet performs feature enhancement strategy by leveraging level-wise information and employing context selection mechanism. In exploiting contextual correlation feature of the crop images our proposed approach demonstrates the effectiveness of the context selection mechanism. Our proposed scheme achieves 88.72% accuracy and outperforms the existing approaches. Our model is evaluated on the cocoa bean dataset constructed from the real cocoa bean industry scene in Indonesia.

## 1. Introduction

Recently the population of human race has surpassed 8 billion people. Food demand is always increasing, resulting in more effort required in producing more ingredients. As the main source of food, agriculture industry must boost its output by effectively using available resource to fulfill this demand. Machine learning approaches, particularly deep learning, are making their way to help solve this problem by improving the agriculture industry in more than one way.

### 1.1. Background

Advancements of technology in the recent years have brought significant changes for various different fields, with agriculture being one of them. Implementation of technology in farming can be found in smart farming and precision agriculture. Smart farming implements technology into automating tasks into most aspects in farming. Precision agriculture involves establishing more control into the practice of farming with the help of technology, such as remote sensing, ripeness classification, pest detection, drought prediction, yield prediction, and crop diversity detection.

For decades, machine learning algorithms had been implemented in both smart farming and precision agriculture, for example in regression and image processing. For the last decade, the deep learning revolution is abruptly changing several areas in both, enabling new level of automation and control. In a recent research on the applying technologies for agricultural sector, the development of smart scheme and the adoption of the current state-of-the-art of the advanced technology to this sector is becoming emerging research trends. For instance, Rezk [1] proposed an Internet of Things (IoT) based scheme combined with the pattern recognition to construct a platform for smart farming applications (SFA). Using this method, the productivity of crop can be enhanced and the environmental causal that might affect the crop production is also predicted. On the other hand, an IoT scheme based on LoRa communication applied to the Indonesian farming area was also presented [2]. Towards the implementation of technological advance, smart farming is even becoming better and reliable for enhancing the productivity and sustainability of our agriculture. Precision agriculture for object recognition, disease detection, smart irrigation can be accurately performed with the guidance and employment of current technology [3].

Several employments of computer vision based schemes in agriculture was also introduced recently [4,5,6,7,8,9]. Furthermore, the deployment of the computer vision is one of the ways to construct an automation scheme for the smart farming application (SFA). For example there are methods implemented for the ripeness classification [10,11,12]. Detecting ripeness of the yield can help farmers decide best time for harvest and also in the harvesting process itself. Other place would be in sorting and packaging the products. By automating packaging or sorting part of the process, production cost can be kept low. Along the ripeness of the crop, specific conditions of the produce can also be monitored by using object detection algorithm [13,14,15]. Particularly, the health of the vegetation or its yield is in general interest, whether the degradation is caused by pest, parasite, or environmental factors.

On the other hand, in recent years image-based object classification has been researched extensively in the machine learning area. One of the most extensively utilized neural network architecture is ResNet [16]. ResNet based image classification schemes have been implemented in many different fields of applications such as medical [17,18,19], geography [20], geology [21], marine engineering [22], and military [23]. Examples of computer vision schemes in smart farming and precision agriculture listed in the previous paragraph also utilize ResNet in their architectures.

It can be seen that image-based classification systems hold important roles in current smart farming and precision agriculture applications. Diverse tasks such as harvesting ripe crops, picking out substandard fruits, detecting sickness or bad conditions of plants, can be done by using an object classification system. However, a system require a robust architecture. Current classification systems are not perfect and always require accuracy improvements. In this work we propose a method to improve the accuracy of neural network classification system, particularly in the field of agriculture. We propose a method by using unique contexts that proliferates in the agricultural products. However, by modifying specific parts, this particular object classification algorithm can also be used in many other disciplines such as health, security, production, supply chain, and others. In the future we aim to adapt our method to other fields that we deemed are suitable for our proposed method to generate most favorable result.

### 1.2. Related Works

By leveraging the efficacy of Convolutional Neural Networks (CNNs), there are abundance attempts to demonstrate the feasibility of CNN based algorithm to achieve good results for SFA task [7,24,25,26,27,28,29,30]. Ref. [7] proposed the use of deep learning approach to conduct an experiment for fruit classifications. Hossain [7] proposed that the employment of CNN based architecture can provide a robust classifier model for the classification tasks and the performance of fruit classifications is improved compared to the capability of traditional approaches. Furthermore, the study of CNN based algorithm performance was also presented by Bai [24], which proposed the scheme for the cocoa bean classifications called progressive contextual excitation (PCE) network to improve the accuracy reached by the traditional model studied by Adhitya [31].

The benefits of using computer vision to agriculture were also presented by the development of Unmanned Aerial Vehicle (UAV) by Jinya [32], which proposed the use of UAV images for monitoring scheme. The perception from the aerial images are analyzed using U-Net based deep learning architecture to detect the wheat disease called wheat yellow rust. The advantages of computer vision approaches for UAV employed in agricultural field were also mentioned in several papers [33,34,35,36]. Deep learning approaches play as an essential approach to achieve the precised monitoring and automation mechanism particularly on the deployment of UAV for agricultural sector [33].

In terms of constructing an accurate model, it is clearly illustrated that leveraging the computer vision based on CNN approach algorithms brings advantages for realizing a powerful and reliable precision agriculture scheme. The proposed work presented in this paper is the effort to improve the efficacy of the authors’ previous work by enhancing the low and high level features retrieved from the backbone network as shown in [24]. We try to further improve the model by utilizing the multi-level correlation between features generally found in the dataset of the crop commodities. Our proposed model is made with the assumption that by employing the context available between these multi-level features we can add more information to the architecture. By exploiting this characteristic, we believe we can obtain a better approach to be used in the development of the smart farming framework compared to currently available models. Moreover, we also adopt a selection algorithm to eliminate unwanted correlation between features and pick out the most prominent correlations. Furthermore, we also study to construct a secure deep learning scheme for the deployment in smart farming scenarios. To the best of our knowledge, this is the first attempt to build a framework for a smart agriculture framework incorporating a secure deep learning scheme.

Research gap that we aim to answer is how to further improve the accuracy of the classification model. To achieve this we utilize the widely implemented neural network image classification architecture, ResNet, and employ a novel algorithm that utilizes correlations of multi-level features generated from the baseline architecture. This proposed method is made specifically to classify agricultural products which tends to possess particular textures, but also suitable for many other applications. From the above elaborations, we can enlist the main contributions of the proposed approach in this paper as follows:

1. We introduce a feature enhancement strategy using context adaptation mechanism by reconstructing deep features from multi-level dependencies to give more accurate feature representation in the SFA task.
2. We devise a method to select most effective contexts to obtain best results. This is done to leverage level-wise information by applying a context selection mechanism. Using the information from the selected contextual representation, an effective approach for discriminating fine-grained categories is performed.

Our paper is further written as follows: Section 2 illustrates our proposed work for the accuracy enhancement called Selective Context Adaptation Network (SCANet). The detail of experimental results is presented in Section 3. The following discussions of our findings are elaborated in Section 4. Section 5 is the summary of the proposed work, experimental findings, and the possible future works from our approach.

## 2. SCANet: Selective Context Adaption Network

In this work we propose a model which utilizes the multi-level correlation between features found in the input images. Images of crop commodities often include certain textural pattern. Our proposed model makes use of the fact that current neural network models utilize several layers in their algorithm. Each layer extracts different level of features from the input. In our work we utilize ResNet architecture to extract the features to each layer. These features can then be associated with each other and from the correlations additional information can be further extracted. The obtained information can be used to improve the result of the algorithm. We eliminate information with inferior value and only select several prominent information that we deem useful to further improve the accuracy.

**Figure 1:**
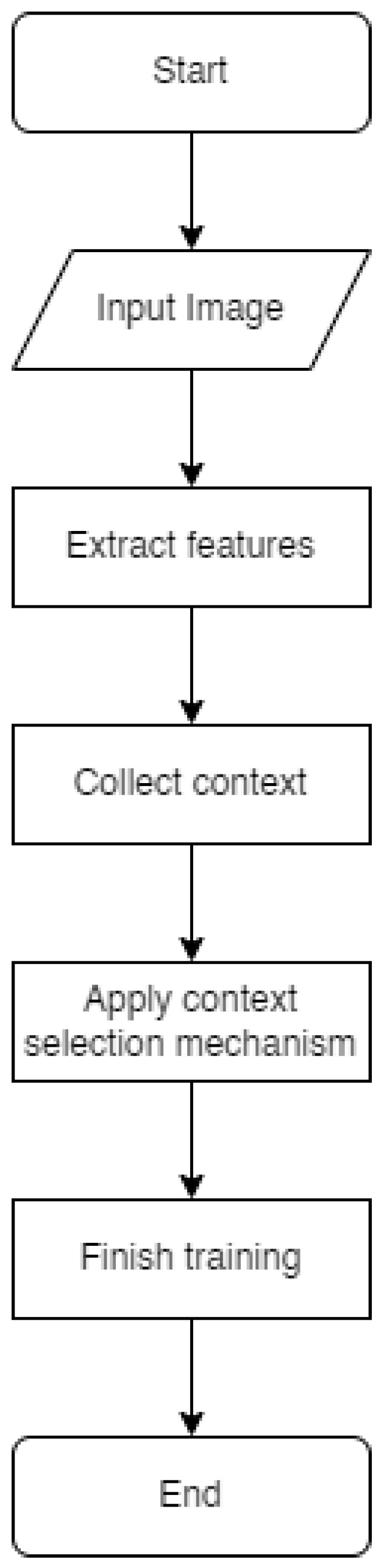
Flowchart of our proposed Selective Context Adaptation Network (SCANet).

**Figure 2:**
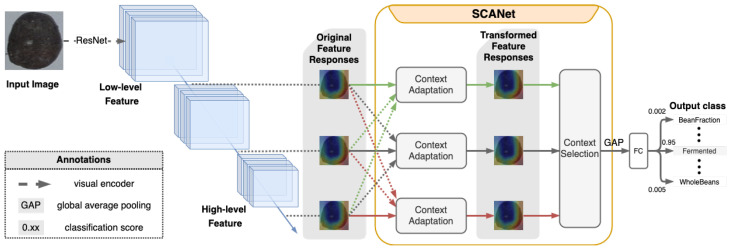
Our proposed Selective Context Adaptation Network (SCANet).

As can be seen in Figure 1, we extract the features using a neural network, collect visual representations of the image from each level, and consider the contextual relationships between these features. Selective Context Adaptation Network (SCANet) receives multi-level feature responses extracted from an input image. Then, the context adaptation mechanism reconstructs deep features by concerning multi-level dependencies. We further apply a context selection mechanism to adaptively modify transformed context to leverage level-wise information. With the selected contextual representation, the final linear transformation layer is able to distinguish fine-grained categories. This flow can be seen in Figure 2, and the flowchart in Figure 1.

### 2.1. Preliminaries

Consider the Smart Farming Application (SFA) task conducted on a set of cocoa bean categories C, where C denotes different types of cocoa beans as described in [31]. Given a fine-grained image, we follow [24] to extract the deep features via the visual extractor of ResNet-50 [16]. Next, we collect multi-level visual representations ${\left\{F_{l}\right\}}_{l=2}^{4}$ as [24] owing to the rich subtle and abstract information from the low level to the high level, where *l* represents the level of visual features.

### 2.2. Selective Context Adaptation Network (SCANet)

Figure 2 illustrates our SCANet by measuring multi-level dependencies from low-level to high-level features for better tackling the fine-grained SFA task, where the visual differences among the cocoa beans are subtle. The quality of visual representation is critical for the smart farming application task. Toward distinguishing fine-grained cocoa beans categories, enhanced contextual representations enable the final linear transformation layer to discriminate similar categories benefited from the rich detailed information of low-level features. PCE collects contextual channel-wise attention via a global-average-pooling operation and further leverages these attentions to re-weight the visual representation at the final layer. However, the global pooling over the entire pixels spatially may introduce global noises potentially. We revisit the context exploration by employing the non-local block [37,38] spatially over multi-level representations. Observed that rich detailed patterns within the low-level features and abstract information from high-level features, we embed the visual representations from other layers to the *l*th layer alternately. Precisely, instead of directly applying the non-local block on each visual representation at the *l*th layer, we reconstruct the level-wise feature concerning other features at different levels via the non-local block to explore the inter-level relationship. We show the positive contributions with our SCANet in the experiments for constructing robust visual representations with respect to contextual semantics in tackling the fine-grained SFA task.

#### 2.2.1. Attention

The attention mechanism [37,38] aims at reconstructing the input feature concerning the pixel-wise similarity, i.e., the affinity matrix, within the feature maps. The affinity matrix estimates the element (pixel) dependency through its key K and query Q and then re-weights value V to highlight similar elements while suppressing dissimilar ones simultaneously. In a nutshell, the basic attention operation *f* is defined as:

(1) $$f\left(Q,K,V\right)=\mathrm{softmax}\left(QK^{T}\right)V\;,$$

where $Q,K,V\in R^{s\times d}$ are embedded *d*-dimensional features with *s* elements.

#### 2.2.2. Context Adaptation Mechanism

This step is responsible for integrating multi-level representations among different levels. As higher level features possess powerful semantics while suffering insufficient detailed information. In contrast, lower level features have rich subtle patterns beneficial for the fine-grained classification. We thus apply a convolution operation to integrate different level features. Please note that we make the spatial dimensions consistent via the bilinear interpolation to eliminate the alias effect as [39] before adopting the convolution operation. Briefly, we define the basic convolution operation Ψ as:

(2) Ψ(X;k,o)=(WX+b),

where *X*, *k*, and *o* indicate the input visual representation, the kernel size of the convolution layer, and the output channels, respectively. W and b separately represent the learnable weight and bias. Consider that a high-level feature, i.e., $F_{4}\in R^{7\times 7\times 2048}$, suffering insufficient detailed information, we leverage lower level features with rich subtle patterns to embed into the high-level feature. Precisely, we first fuse low-level features, i.e., $F_{2}\in R^{28\times 28\times 512}$ and $F_{3}\in R^{14\times 14\times 1024}$ as:

(3) $$\begin{array}{cc} {\bar{F}}_{2} & =\mathrm{interpolation}\left(\Psi \left(F_{2};1,2048\right)\right)\;\in R^{7\times 7\times 2048}\;, \end{array}$$

(4) $$\begin{array}{cc} {\bar{F}}_{3} & =\mathrm{interpolation}\left(\Psi \left(F_{3};1,2048\right)\right)\;\in R^{7\times 7\times 2048}\;, \end{array}$$

(5) $$\begin{array}{cc} S_{4} & =\Psi \left({\bar{F}}_{2}\|{\bar{F}}_{3};3,2048\right)\;\in R^{7\times 7\times 2048}\;, \end{array}$$

where ${\bar{F}}_{2}$ and ${\bar{F}}_{3}$ in Equations (3) and (4) both represent interpolated feature maps to make the spatial dimensions consistent. The symbol ‖ in Equation (5) denotes the concatenation operation along the channel dimension. $S_{4}$ collects detailed information from low-level features and serves as a supported feature to enhance the high-level feature $F_{4}$. The enhanced high-level feature $\hat{F_{4}}$ leveraging the supported feature $S_{4}$ via Equation (1) is formally defined as:

(6) $$\begin{array}{c} {\hat{F}}_{4}=f\left(S_{4},F_{4},F_{4}\right)\in R^{7\times 7\times 2048}\;. \end{array}$$

Analogously, the middle-level feature $F_{3}$ concerning low-level feature $F_{2}$ and high-level feature $F_{4}$ is formulated as:

(7) $$\begin{array}{cc} {\bar{F}}_{2} & =\mathrm{interpolation}\left(\Psi \left(F_{2};1,1024\right)\right)\;\in R^{14\times 14\times 1024}\;, \end{array}$$

(8) $$\begin{array}{cc} {\bar{F}}_{4} & =\mathrm{interpolation}\left(\Psi \left(F_{4};1,1024\right)\right)\;\in R^{14\times 14\times 1024}\;, \end{array}$$

(9) $$\begin{array}{cc} S_{3} & =\Psi \left({\bar{F}}_{2}\|{\bar{F}}_{4};3,1024\right)\;\in R^{14\times 14\times 1024}\;, \end{array}$$

(10) $$\begin{array}{cc} {\hat{F}}_{3} & =f\left(S_{3},F_{3},F_{3}\right)\in R^{14\times 14\times 1024}\;. \end{array}$$

#### 2.2.3. Context Selection Mechanism

**Figure 3:**
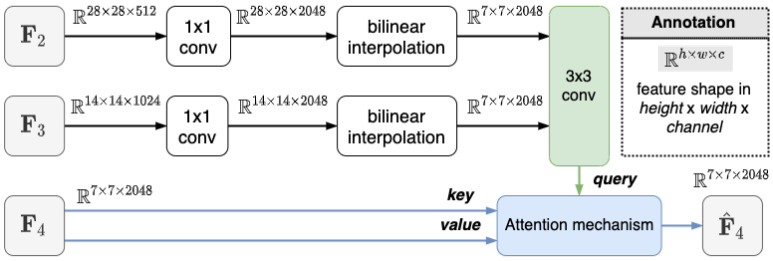
Diagram of Context adaptation mechanism.

Once the feature has been extracted and constructed, therefore, we adopt the context selection scheme. Basically, the context selection mechanism aims to dynamically select meaningful enhanced features which are beneficial for the following fine-grained category discrimination task. Then, the enhanced features constructed by the level-wise information is selected to perform a classification task. The entire diagram of our approach is illustrated in the Figure 3.

## 3. Performance Results

### 3.1. Dataset

#### Cocoa Bean Images

**Figure 4:**
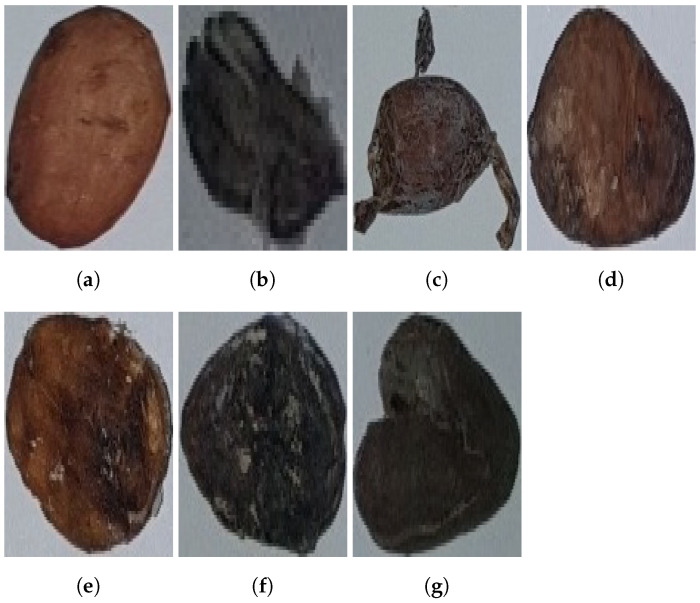
Cocoa Bean Image collected from Indonesian cocoa bean factory, Sulawesi, Indonesia [31]. (**a**) Whole Beans. (**b**) Beans Fractions. (**c**) Skin-Damaged Beans. (**d**) Fermented Beans. (**e**) Unfermented Beans. (**f**) Moldy Beans. (**g**) Broken Beans.

In this experiment, we tested our approach using the same cocoa bean dataset as presented by [31]. This dataset is not available for public. There are 7428 images divided into 7 categories such as: (1) whole beans, (2) Beans fractions, (3) skin-damaged beans, (4) fermented beans, (5) unfermented beans, (6) moldy beans, and (7) broken beans. Furthermore, the dataset is randomly divided into 75% for training, 15% for validation, and 10% for testing. We implemented this distribution for each categories. The data were taken from the real environment using a compact digital camera and sampled in the factory. Therefore, the collection of the data is further processed by sorting based on the Indonesian Standardization Institution especially following the rule for exporting quality [31,40]. The sample of the data is shown in Figure 4 and the distribution of each class is presented in Table 1.

### 3.2. Implementation Details

ResNet-50 [16] is adopted as the baseline and constructed as the visual encoder. The training stage for the visual encoder is performed without loading any pre-trained weight. Bilinear interpolation is used to create 224×224 pixel images as the input images. The initial learning rate is $1\times 10^{-2}$ and decayed by $5\times 10^{-4}$. The visual encoder is trained from scratch using SGD optimizer and a batch size is 128.

### 3.3. Ablation Study

In order to have an understanding related to the each feature map, we conducted ablation study. The aim of the ablation studies here is to investigate the features constructed from each level of features. Therefore, we have insight of the performance for the involvement of each feature map constructed by each layer.

As presented in Table 2, we perform ablation studies on each level of the SCANet layer. The baseline of this study is ResNet-50 as the original network then we are further enhancing the base network using the proposed schemes. As explained that ${\bar{F}}_{2}$ and ${\bar{F}}_{3}$ represent the interpolated feature maps to be used for the enhancement of ${\bar{F}}_{4}$, Table 2 shows that using the feature from ${\bar{F}}_{4}$ alone, the accuracy is 85.71%. By additional features from ${\bar{F}}_{2}$ and ${\bar{F}}_{3}$, the overall accuracy can be further enhanced and reaching 88.72%. In addition, we also conducted a study using different context selection schemes as shown in Table 3. It is clearly seen that our proposed scheme is superior compared to other selection methods.

### 3.4. Comparison with Existing Works

To compare our approach with existing similar works, we list several existing works in Table 4. The performance comparison is conducted by using the top-1 accuracy metric represented by the formula

(11) $$\begin{array}{c} \frac{TP+TN}{TP+TN+FP+FN}\;. \end{array}$$

where TP, TN, FP, and FN in Equation (11) are the representative of true positive, true negative, false positive, and false negative, respectively.

As depicted in Table 4, our approach which can attain 88.72% accuracy outperforms existing works. By utilizing the context selection mechanism strategy, we can achieve better performance compared to our previous work called PCENet. In addition, SCANet also eliminates the post-processing enhancement strategy as proposed in [31] and shows the superiority compared to other existing studies.

We made further comparison using publicly available datasets of other agricultural based scenarios: Corn, Apple, and Grape Leaf Diseases dataset. By using readily available dataset, we can expand our comparison to include more methods. In this work we include results from DenseNet, Modified LeNet, VGG19 + K-means, AlexNet, SVM, and LS-SCM. In addition to expanding our comparison, by using these datasets we want to evaluate how our proposed model handles more diverse agricultural-related scenarios. As with the results from the cocoa bean dataset, our proposed model shows superior accuracy compared with existing models. Table 5 shows the results from corn leaf diseases dataset, while Table 6 and Table 7 show results from apple and grape leaf diseases dataset, respectively.

## 4. Discussion

As presented in Table 4, Table 5, Table 6 and Table 7, our approach gives better performance compared to other existing approaches. Compared to the result by [24,31], our proposed scheme produces higher accuracy for the fine grained classification task with 88.72% of which [24,31] only produced 65.08% and 86.09%, respectively. Thus, it is shown that by leveraging a feature selection mechanism, we can improve the performance of the deep learning based approach for image classification especially to construct a smart farming framework.

Our previous study shows that leveraging the contextual channel-wise attention can improve the accuracy of the fine-grained classification task on cocoa bean dataset. However, the construction of global context introduced by such method may include global noises that can destruct the final classification on the fully connected layer. By implementing a context selection mechanism, more accurate results can be produced as presented in current study. One downside of our method is the introduction of interpolation in our design which will invariably increase the size of the resulting inference model.

## 5. Conclusions

In this study, we have demonstrated the improvement of fine-grained classification task for Smart Farming Applications (SFA). Image classification always aim for improvement of accuracy and current researches are trying to utilize different method to achieve this. The context selection mechanism in our proposed scheme called SCANet can enhance and boost the performance to address the fine-grained problems in the SFA. By leveraging and combining the lower level and higher level features, SCANet can have more sufficient and enriched information. Eventually, SCANet processes the constructed features by employing the context selection mechanism and can further enhance the model performance. From the results obtained in this study, SCANet outperforms other existing approaches by reaching 88.72% accuracy on the cocoa bean dataset. It can be seen that our method is ideal for classifying cocoa beans images, and as such we believe it can be utilized to sort cocoa beans in real life process, and additionally also for different types of bean. For future studies, we are trying several improvements: implement compression techniques into our works, add selective environment contextual correlation, and put the inference model in real-life practical test.

## Figures and Tables

**Table 1:** Distribution Class of the Cocoa Bean Images.

Classes	Amount of Images	Training	Validation	Test
Whole Beans	1187	891	178	118
Broken Beans	1046	786	156	104
Bean Fractions	426	321	63	42
Skin-Damaged Beans	822	617	123	82
Fermented Beans	916	688	137	91
Unfermented Beans	1776	1333	266	177
Moldy Beans	1255	942	188	125
Total of the Data	7428	5578	1111	739

**Table 2:** Ablation study of the level transformation mechanism.

$\hat{F_{2}}$	$\hat{F_{3}}$	$\hat{F_{4}}$	Top-1 Accuracy
baseline	baseline	baseline	82.71
-	-	✓	85.71
-	✓	✓	88.72
✓	✓	✓	86.09

**Table 3:** Ablation study of the context selection mechanism.

Context Selection Schemes	Top-1 Accuracy
Average	86.47
Conv1 × 1	87.59
SCANet	88.72

**Table 4:** Comparison of the classification results using cocoa bean dataset. The visual feature enhancement employing GLCM scheme for post-processing [41] is shown by “*”.

Model	Post-Process	Top-1 Accu.
Adhitya’s model (SVM)	no	59.14
Adhitya’s model (XGBoost)	no	56.99
Adhitya’s model (SVM *)	yes	61.04
Adhitya’s model (XGBoost *)	yes	65.08
ResNet-50	no	82.71
PCENet	no	86.09
Compressed PCENet [42]	no	86.09
SCANet (Ours)	no	88.72

**Table 5:** Comparison of the classification results using corn leaf diseases dataset.

Classification Model	Accuracy
VGG19 + K-means [43]	93.4
Modified LeNet [44]	97.89
DenseNet [45]	98.06
SCANet (Proposed model)	98.31

**Table 6:** Comparison of the classification results using apple leaf diseases dataset.

Classification Model	Accuracy
DenseNet [46]	93.71
AlexNet [47]	97.62
SCANet (Proposed model)	99.69

**Table 7:** Comparison of the classification results using grape leaf diseases dataset.

Classification Model	Accuracy
SVM [48]	93.01
LS-SCM [49]	97.66
SCANet (Proposed model)	99.88

## Data Availability

Data sharing not applicable.
